# Maintenance of venomous snakes in captivity for venom production at Butantan Institute from 1908 to the present: a scoping history

**DOI:** 10.1590/1678-9199-JVATITD-2020-0068

**Published:** 2021-01-22

**Authors:** Kathleen Fernandes Grego, Samira Emanuela Maria Vieira, Jarbas Prado Vidueiros, Eliana de Oliveira Serapicos, Cibele Cíntia Barbarini, Giovanni Perez Machado da Silveira, Fabíola de Souza Rodrigues, Lucas de Carvalho Francisco Alves, Daniel Rodrigues Stuginski, Luciana Carla Rameh-de-Albuquerque, Maria de Fátima Domingues Furtado, Anita Mitico Tanaka-Azevedo, Karen de Morais-Zani, Marisa Maria Teixeira da Rocha, Wilson Fernandes, Sávio Stefanini Sant’Anna

**Affiliations:** 1 Laboratory of Herpetology, Butantan Institute, São Paulo, SP, Brazil.

**Keywords:** Snake, Venom, Snake husbandry, Venom production

## Abstract

Maintenance of snakes at Butantan Institute started in the last century, intending to produce a different antivenom serum to reduce death caused by snakebites. Through a successful campaign coordinated by Vital Brazil, farmers sent venomous snakes to Butantan Institute by the railway lines with no cost. From 1908 to 1962, the snakes were kept in an outdoor serpentarium, where venom extraction was performed every 15 days. During this period, the snake average survival was 15 days. In 1963, the snakes were transferred to an adapted building, currently called Laboratory of Herpetology (LH), to be maintained in an intensive system. Although the periodicity of venom extraction remained the same, animal average survival increased to two months. With the severe serum crisis in 1983, the Ministry of Health financed remodeling for the three public antivenom producers, and with this support, the LH could be improved. Air conditioning and exhausting systems were installed in the rooms, besides the settlement of critical hygienic-sanitary managements to increase the welfare of snakes. In the early 1990s, snake survival was ten months. Over the years to the present day, several improvements have been made in the intensive serpentarium, as the establishment of two quarantines, feeding with thawed rodents, an interval of two months between venom extraction routines, and monitoring of snake health through laboratory tests. With these new protocols, average snake survival increased significantly, being eight years for the genus *Bothrops*, ten years for genus *Crotalus* and *Lachesis,* and four years for the genus *Micrurus*. Aiming the production of venoms of good quality, respect for good management practices is essential for the maintenance of snakes in captivity. New techniques and efficient management must always be sought to improve animal welfare, the quality of the venom produced, and the safety of those working directly with the venomous snakes.

## Background

Between 1895 and 1897, the newly graduated doctor Vital Brazil, from Campanha city, Minas Gerais state, moved to Botucatu, São Paulo state. It is not possible to specify the exact date of his arrival, although it is known that in the early days of 1897 he practiced and travelled from farm to farm, caring for his patients [1]. Vital Brazil, impressed with the number of patients bitten by venomous snakes, decided to seek a medicine that could save lives. He started his studies evaluating the effectiveness of plant strata that tradition pointed out as a solution to the problem [1]. In 1898, already working at the Bacteriological Institute of São Paulo, he prepared the first antivenom of proven efficacy against the envenomation by *Bothrops* and *Crotalus* [1]. In 1899, the director of the Adolfo Lutz Institute suggested to the State Governor the creation of the Serumtherapy Institute of the State of São Paulo, which, after its completion, was directed by Vital Brazil [2,3]. At that moment, the activities of the current Butantan Institute were initiated, which became official in 1901. The use of snake venom to treat envenomation by snakes was first discovered by Albert Calmette, a French physician. This scientist believed in a *universal* antivenom produced only with the *Naja* sp. venom [2,4]. Vital Brazil, founder of Butantan Institute, began to keep snakes in captivity for venom extraction for the production of antivenom serum. As a consequence, he discovered the worldwide *specificity* of antivenoms [1,3]. Snakes arriving at Butantan Institute were used to supply venom for the immunization of horses that produced antivenom sera; for chemical, pharmacological and immunobiological researches; and to provide laboratories with material for the study of snake biology and systematics [5]. 

Snakes arrived at Butantan Institute due to a program coordinated by Dr. Vital Brazil called “The defense against ophidism” [3,6], which aimed to disseminate information to the general public about the new treatment against snake bites, mainly in rural areas [6,7]. With this program, Vital Brazil attracted the interest of farmers and rural workers, establishing a system of exchanging venomous snakes for antivenom serum vials, as well as all material necessary for its application; encouraging the population to send snakes inside safe wood boxes by the rail lines, free of charge [6,7]. As a result, the institute received snakes in a quantity sufficient to manufacture antivenom serum on a scale compatible with the demands of the population [6,7]. Each snake received was recorded in books, with the name of the donor, the snake species, gender, locality of origin and date of arrival. Since Butantan Institute’s foundation in 1901 until 1977, about 1.1 million snakes were received, 80% of which were from venomous species [8]. As a result of this successful campaign at the beginning of the last century, still today the Institute receives a great variety of snakes, although we do not encourage their capture, and for a long time do not exchange antivenom serum for snakes. Nowadays the Institute receives snakes mostly from São Paulo State.

Based on bibliographic references and database records from the Laboratory of Herpetology (LH) concerning the maintenance of snakes at Butantan Institute; we describe the maintenance of snakes for venom production in four different periods, showing how the changes done in captive husbandry impacted the welfare of venomous snakes and the quantity of venom produced.

## Maintenance of Snakes from 1908 to 1962

**Figure 1.**
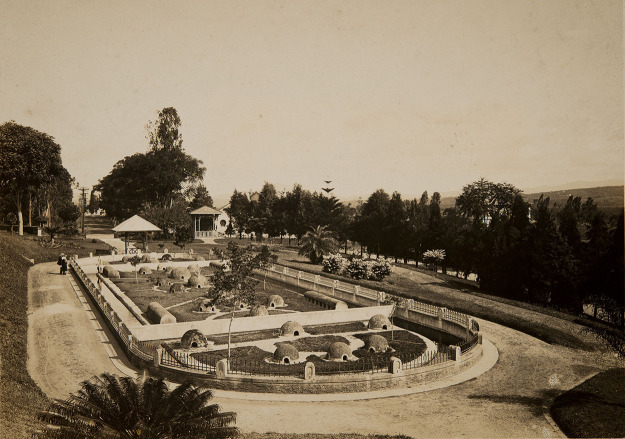
View of the semi-intensive serpentarium in 1926 (photo courtesy of the Memory Center Collection of Butantan Institute).

During this period, snakes were kept in a semi-intensive serpentarium measuring 500m^2^ [5]. Venom extractions were performed publicly, becoming an attraction for the general public. This outdoors serpentarium had shelters in the shape of igloos to protect snakes from sun, cold and rain; shallow ponds; and in this space several demonstrations about handling snakes and prevention of snake bites were realized (Figure 1). Venom extraction was performed every 15 days [9], and despite the caprice in its conception, the semi-intensive serpentarium did not provide snakes with good living condition [10]. At this period, snakes’ mortality percentage was about 92 to 98% yearly, meaning that each snake was extracted only 0.6 to 1.5 times [9]. 

In this type of maintenance, the survival rate of snakes was about 15 days. The low survival rates in the outdoors serpentarium from its foundation to 1999 was probably due to stressful factors, including the impossibility to thermoregulate efficiently, the lack of controlled feeding protocol, the high population density, associated with the absence of hygienic-sanitary and preventive medicine [11].

## Maintenance of Snakes from 1963 to 1987

While the demand for antivenom serum increased steadily, the number of snakes received at the Institute remained the same [9]. In order to increase venom production, the veterinary Hélio Belluomini (head of the Experimental Animal Facility of Venomous Snakes - current Laboratory of Herpetology) began maintaining snakes in an intensive serpentarium, introducing several changes in the snakes’ husbandry environment to prolong their survival rate. Belluomini also studied their biology in captivity and increased the number of extractions/animal [8,9,12]. In contrast to semi-intensive systems, intensive serpentariums have the advantage of controlling abiotic factors, such as temperature, humidity, and photoperiod, which facilitates the maintenance of species with different husbandry requirements; allow greater individual control concerning feeding and diseases; besides optimizing space [10,13,14].

Some of the implemented changes were the heated rooms with electrical resistances; the maintenance of up to four snakes in each wooden box, and the supply of rodents two days after venom extraction [9]. Until 1978, the extraction periodicity was 15 to 20 days and snakes’ average survival for about two months. In the mid-70s, the use of carbon dioxide (CO_2_) was adopted in the indoors serpentarium to aid in the venom extraction routine, as a short-term anesthesia. The inhalation of pure CO_2_ for four to ten minutes is a safe and not traumatic method for snakes, facilitating venom extraction and making it more secure to the staff. Carbon dioxide gas reduces the risk of accidents significantly, without affecting the survival of animals or venom production [15].

From 1979 to 1987, although venom extraction periodicity has been extended to 30 days, snake survival remained the same, an average of two months. It is noteworthy that the survival rate of snakes maintained in the indoor serpentarium was higher than the survival rate of animals maintained in the outdoor one. Consequently, venom production also increased under intensive care (Table 1). Nevertheless, much more was needed to improve snakes’ welfare in captivity.

**Table 1.** Venom yielded per snake [mean volume (mL) ± standard variation], according to the management and year of maintenance.

	Period	Crotalus durissus	Bothrops jararaca
Venom yielded/snake in the outdoor serpentarium	1908-1962	0.18 mL*	0.10 mL*
Venom yielded/snake in the indoor serpentarium	1963-1968	0.26 mL*	0.20 mL*
	2000-2004	0.30 mL*	0.27 mL*
	2005-2019	0.45 ± 0.11mL	0.46 ± 0.18 mL
Venom yielded/recently wild-caught snake in the screening room	2005-2019	0.19 ± 0.07 mL	0.20 ± 0.09 mL

* In these periods, our database has only the mean value of venom extracted/animal.

## Maintenance of Snakes from 1988 to 2000

Until 1985, antivenom serum was not included in the National Immunization Program of the Health Ministry. During those years, Brazilian antivenom was produced by Butantan Institute (São Paulo), Vital Brazil Institute (Rio de Janeiro), and Ezequiel Dias Foundation (Minas Gerais), besides Syntex do Brazil, a private pharmaceutical company [16]. The national antivenom production and distribution entered into a crisis when Syntex do Brazil closed its immunobiological production in 1983 [17]. To solve the shortage of antivenom serum, in 1986 the Ministry of Health provided funds to improve and modernize methods and processes of the three public producers, proposing to fully acquire all their production [16,17]. Hence, from the second semester of 1987 to 1989, the intensive serpentarium could be remodeled and its infrastructure improved. A significant renovation was installing an air system to control the relative humidity and temperature inside maintenance rooms, in addition to an efficient ventilation system. Furthermore, the biologist Wilson Fernandes, head of the LH at this period, individualized snakes in wooden boxes; maintained venom extraction once per month with the aid of CO_2_; changed feeding protocol (the snakes were fed with rodents one week after extraction routine); and instituted hygienic-sanitary and prophylactic medicine, such as deworming, and quarantine of the animals. These enhancements improved the maintenance and welfare of venomous snakes in captivity in such a way, that from 1989 to 1991 the average survival of snakes was five months, and in 1992, the survival increased to ten months.

In the 1990s, the maintenance of *Micrurus corallinus* and *M. frontalis* at LH began. Keeping these species in captivity was a challenge, due to the difficulty in adapting them to captive husbandry and to their specific diet, which is based on amphisbaenians and snakes [18,19]. During this period, the average survival rate of coral snakes was less than five months [18,20].

## Maintenance of Snakes from 2000 to date

Maintenance of snakes at LH from the year 2000 to date, has not changed much, although improvements are continuously being made to increase snakes’ welfare and the safety of those working directly with them.

Nowadays, LH maintains approximately 1000 specimens of venomous snakes, including coral snakes (*Micrurus corallinus, M. frontalis, M. ibiboboca, M. spixi* and *M. altirostris),* bushmasters (*Lachesis muta*)*,* pit vipers (*Bothrops jararaca, B. jararacussu, B. alternatus, B. moojeni, B. neuwiedi, B. mattogrossenssis, B. pauloensis, B. pubescens, B. marmoratus, B. erythromelas, B. insularis, B. fonsecai, B. cotiara, B. billineata, B. leucurus* and *B. atrox*), rattlesnakes (*Crotalus durissus*), and naja (*Naja kaouthia*)*.* Besides extracting venom, researchers and technicians of LH also study snake reproductive biology, behavior, and husbandry; investigate pathologies affecting specimens in captivity; and study biological, biochemical and pharmacological aspects of their venoms. 

**Figure 2.**
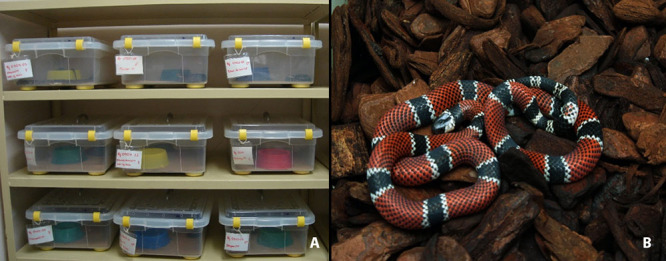
**(A)** Cages used on viperids maintenance with corrugated card box substrate and plastic water pots. Each cage has a badge with the snake code number, locality of origin and gender. **(B)** Bark substrate used in the maintenance of coral snakes (*Micrurus corallinus*).

Snakes are kept in plastic cages with different dimensions according to their size (a coiled snake cannot occupy more than 1/3 of the total area of the cage [14,21]. Cages are made of impermeable transparent plastic material, free from fissures and inert to disinfectants and cleaning chemicals, as recommended by WHO [21], and arranged on shelves to optimize room’s space. The genus *Bothrops* and *Crotalus* are kept in these cages with corrugated cardboard substrate and water freely available (Figure 2A), the genus *Micrurus* is maintained on cages with bark substrate, also with water at will [20] (Figure 2B). In the cage of each snake, there is a badge with the snake’s code, locality of origin, and gender. The genus *Lachesis muta* due to its large size is kept in a 20 m^2^ room, with water within reach, artificial turf, humidifier fan, and shelters with fluffy substrate. Rooms are maintained with temperatures between 23 to 26ºC, with relative humidity around 60% and a light/dark cycle of 12h [22]. The temperature and humidity of each room are monitored with thermohygrometers and are daily noted on spreadsheets.

Since 2015, venom from each snake is extracted every 60 days. Feeding continues to be given every 30 days, one week after routine extraction. Regarding feeding, viperids are fed with rodents (*Mus musculus* or *Rattus norvegicus*) from the Rodent Reproductive Center at Butantan Institute, and since 2015 rodents are euthanized in CO_2_ gas, frozen and given thawed to snakes. The new maneuver avoids the suffering of rodents, as well as possible injuries preys can cause to snakes [23]. Ninety-five percent of our snakes accept promptly thawed preys. Prey is given in a proportion of 10 to 20% of the snake's body weight to avoid obesity, which can spoil the animals' health, cause infertility and even death [23,24]. 

Concerning the feeding of ophiophage coral snakes (*Micrurus corallinus* and *M. frontalis),* young viperids and small dipsadini snakes are euthanized, frozen, and given in a proportion of 30 to 40% of the coral body weight [20]. Frozen preys are thawed in hot water and offered to snakes. 

Some factors may be responsible for anorexia in snakes, such as systemic and parasitic diseases; animals in ecdysis process; low or high temperature and humidity; females that have recently given birth; snakes with neoplasms; among others [23,25]. We verified that during the reproductive season (from April to August), some animals, mainly males, are not interested in feeding, corroborating other works in the area [26,27]. A protocol used at LH is to force-feed animals that refuse prey for three consecutive months and, subsequently, investigate and correct the cause of inappetence. Anorexic snakes are sedated in a recipient with CO_2_ gas and, with the aid of tweezers, thawed rodents lubricated in vitamin complex are gently forced in the snake’s esophagus (the incisor teeth of the rodent must be cut to avoid lesions in the snake’s esophagus). In relation to anorexic elapids, a specific diet made at LH is given through an esophageal tube [20]. 

All improvements done in the indoors serpentarium over the years are responsible for the higher survival rates we have attained nowadays. The average survival rate for the different genus considers snakes born in captivity, as well as newly arrived ones after quarantine period. The average survival rate for the genus *Bothrops* is of eight years, for the genus *Crotalus* and *Lachesis* of ten years, and for the genus *Micrurus,* four years. LH has a team of trained technicians and biologists and at least one veterinarian, as recommended by CONCEA [14]. The facility is composed of one screening room; two quarantines; ten rooms for the maintenance of venomous snakes; a consultation/surgical room; a reproduction room; a nursery room; a clinical analysis laboratory; a pathological anatomy laboratory; and laboratories to process and study snakes’ venoms.

## Screening Room

Venomous snakes arriving at the Institute donated by farmers, Fire Department, or Environmental Police are sent to LH where they stay for a maximum of seven days at the screening room. In this room, all snakes have their venom extracted and some specimens, according to their health status, gender, and locality of origin, are selected to go to the quarantine room. It is important to say that for the production of a high quality antivenom serum, it is essential to have specimens from a wide geographical area [10,21]. Snakes not selected are forwarded to research projects, zoos or zoological collection.

In the screening room, snakes selected to quarantine are clinically examined; weighed and measured [snout-vent length (SVL) and total length (TL)]; receive their first dose of anthelminthic (Ivermectin 0.2 mg kg ^-1^) and are immersed in an ectoparasiticide solution (Trichlorfon at 0.2%). This first prophylactic management is of paramount importance, as incoming specimens from nature usually host a vast range of endo and ectoparasites that can predispose animals to infections, food malabsorption, regurgitation, and even lead them to death if not eliminated [11,23,28].

**Figure 3.**
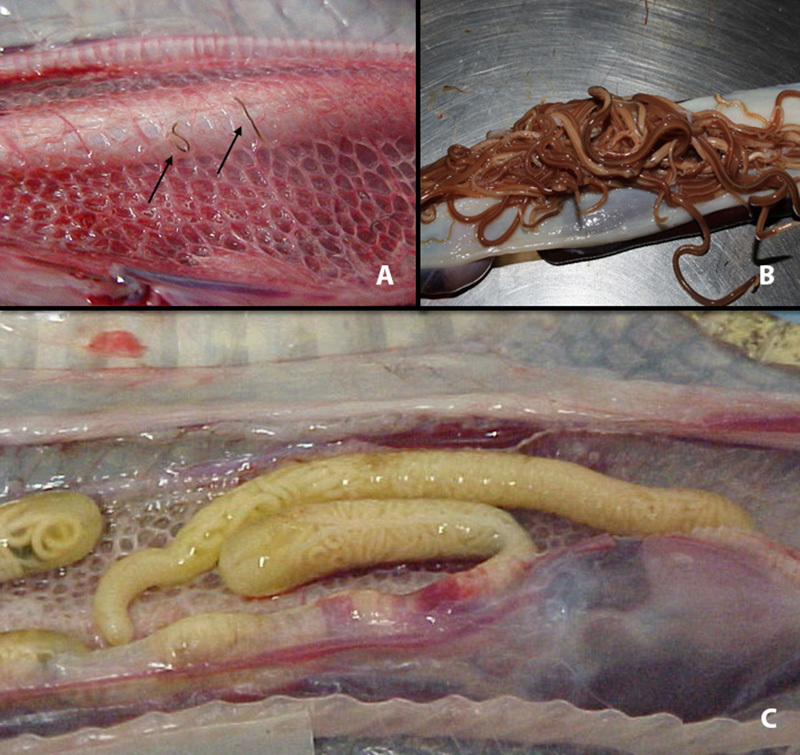
**(A)** Nematode *Rhabdias* sp. in the lung of *Bothrops jararaca*. **(B)** Ascarids in the upper gastrointestinal tract of *Crotalus durissus*. **(C)** Pentastomid parasite in the lung of *Bothrops jararaca.*

The main endoparasites found in new-arriving viperids are pulmonary nematodes (Figure 3A), gastrointestinal nematodes (Figure 3B) and pulmonary pentastomida (Figure 3C). More rarely gastrointestinal cestodes and trematodes are found [11,23,28]. Protozoa are also common, such as flagellates and coccids. Among ectoparasites, the main ones are ticks (*Amblyomma* sp.) and mites (*Ophionyssus natricis*).

After the prophilactic procedures described above, snakes are sent to the quarantine room strategically located next to the screening room but separated by a door (physical barrier). Each snake receives an Individual Form where information about its locality of origin; date of arrival; gender; SVL - TL and weight; specific code number; microchip number; date of feeding; date of ecdysis; date of venom extraction; veterinary treatments; and date of pairing, gestation and parturition, are annotated.

## Quarantine room

The introduction of incoming animals in a new place produces stressful responses that if prolonged can lead to distress, exhaustion, and even death [23,29,30,31]. Therefore, the animals must go through a period of acclimatization (quarantine) before being introduced into the maintenance rooms. Moreover, quarantine of incoming snakes is essential to prevent the spread of infectious and parasitic diseases [22,31,32]. 

The LH works with two quarantine rooms (Q1 and Q2) that are close to the screening room and to the Maintenance rooms, but separated by them by physical barriers as recommended by WHO [21]. Q1 receives animals weekly and is the beginning of the acclimatization. In this room, the snakes are inspected daily, receive the second dose of anthelminthic (15 days after their arrival date), and are monthly fed. Q2 receives healthy snakes from Q1 in an “*all-in, all-out*” system. In this system, healthy snakes that are feeding appropriately and are at least 30 days in Q1 are transferred in the same day to Q2, where they stay isolated for 30 days. After this period, they are all transferred to the Maintenance rooms in the same day. 

While in Q2 the animals are daily inspected and if an individual dies, the period is extended for more 30 days. In this case, the cause of death is investigated. Therefore, our quarantine period lasts for at least 60 days. Only snakes with negative coproparasitological exams are transferred to the Maintenance rooms. Venom extraction is not performed in the quarantine rooms. 

## Maintenance rooms for venom production

At the entrance of the Maintenance rooms’ hall, there is a small area of 8 m^2^ where technicians wear aprons and boot/shoe covers to avoid external pathogens into the animal facility. Maintenance rooms have 20m^2^ with epoxy coated walls and granulite floor for easy cleaning. Each room has the capacity to maintain 90 snakes in individual plastic cages arranged in shelves. All rooms have a thermohygrometer for daily temperature and humidity monitoring, as well as a programmable light timer to guarantee light/dark cycles. Snakes are inspected daily, and cages are changed whenever necessary. In each Maintenance room we maintain snakes of the same species or snakes with similar husbandry requirements. Venom extraction is performed monthly inside the room, with the aid of carbon dioxide gas (CO_2_) and a stainless steel table. The snakes are placed in recipients saturated with CO_2_ gas, where they remain for about 5 minutes. After cessation of stimuli, the snakes are removed from the recipient and venom extraction performed by massage of venom glands, which takes about 5 to 8 seconds. The use of CO_2_ does not cause any harm to snakes [33].

**Figure 4.**
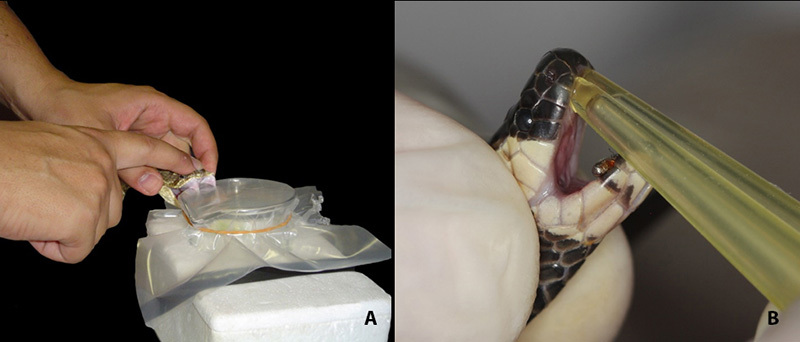
**(A)** Venom extraction in *Crotalus* sp. performed in a glass Beaker covered with a thick plastic sheet, immersed in ice bath. **(B)** Venom extraction in coral snake performed with tips connected to the proteroglyphous fangs.

Viperid venom extraction (*Bothrops* sp.*, Crotalus durissus and Lachesis muta*) is carried out in a glass Beaker covered with a thick plastic, immersed in an ice bath. The plastic is exchanged after each extraction to avoid contamination between individuals (Figure 4A). After the extraction procedure, chlorhexidine at 0.12% is sprayed in the fangs’ sheaths to prevent infections from micro lesions that can occur during the extraction procedure. The snake is then weighed, measured (SVL-TL) and returned to its cage. One week after the extraction, snakes are fed with thawed rodents.

Elapid venom extraction (*Micrurus* sp.) is differentiated. Due to the small size of these snakes and their reduced venom glands, pilocarpine is administered intradermally (10 mg kg ^-1^) ten minutes before venom extraction to increase milking efficiency [34]. Pilocarpine is a natural alkaloid with cholinergic agonist activity that binds to muscarinic receptors inducing secretion from exocrine glands [35]. Using pilocarpine we observed an increase of 127% in the volume of venom extracted from *Micrurus corallinus,* with no significant change in venom composition [34]. Coral snakes’ extraction is performed with tips connected to the proteroglyphous fangs (Figure 4B), and, with the aid of pipettes, the venom is transferred to microcentrifuge tubes in ice bath. One week after extraction, coral snakes are fed with thawed snakes in a proportion of 30 to 40% of their body weight [20] for three alternate weeks.

All rooms are equipped with personal protective equipment (PPEs) and tools necessary for the handling of snakes, such as hooks, Lutz’s Loop, restraining tubes, snake tongs, and tweezers, which are immersed in a 4% sodium hypochlorite solution after use.

## Consultation/Surgical Room

In this room strategically built near the maintenance rooms, the veterinary performs clinical examination, ultrasound exams, differential diagnoses, and prescribes treatments to be established in snakes presenting symptoms of disease or altered behavior, as respiratory distress, skin lesions, gastroenteric disorders, including others. Ill animals and the ones in treatment are not submitted to venom extraction. Surgeries are also performed in this room whenever necessary. The furniture of this room consists of a stainless steel table, an ultrasound machine, an inhalation anesthesia machine, a medicine cabinet, a sterilization oven for surgical materials, and a refrigerator for medicines.

## Reproduction Room

**Figure 5.**
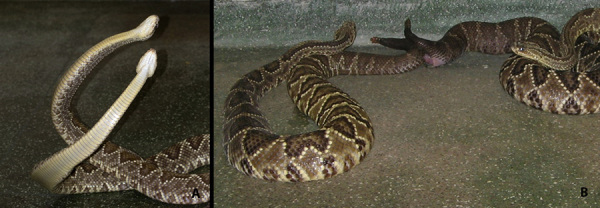
**(A)** Combat bout in *Crotalus durissus* (rattlesnake) males. **(B)** Mating in *Crotalus durissus.*

**Figure 6.**
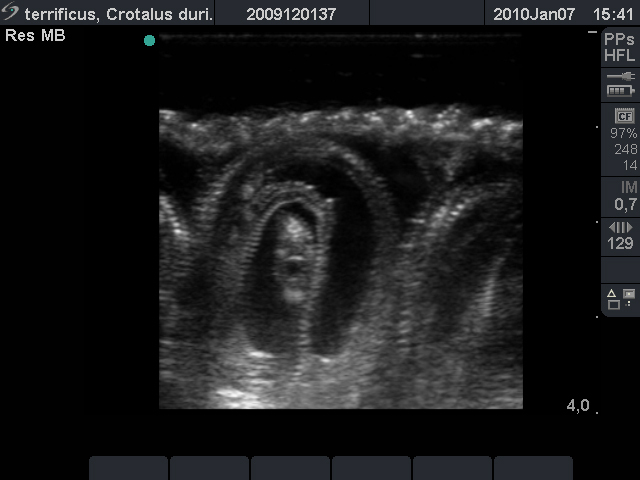
Ultrasound image of *Crotalus durissus* fetuses.

The reproduction room has no furniture, but four cameras strategically placed to record reproductive behavior, such as combat bouts in some species (Figure 5A), male courtship, and mating (Figure 5B). We usually place two males in the room to increase competitiveness and reproductive interest, and after 30 minutes a female is placed in the room. The day after pairing, a vaginal smear is performed to check for the presence of sperm. If the result is positive, an ultrasound exam is done monthly to monitor the female’s pregnancy until birth (Figure 6). Brazilian viperids, with the exception of *Lachesis muta*, are viviparous snakes; elapids (coral snakes) and *Lachesis muta* (bushmasters) are oviparous. Due to the decrease in the arrival of snakes at Butantan Institute, we have been improving reproduction to be self-sufficient, depending less on snakes coming from nature. Nowadays, 60% of the snakes maintained at LH are captive-born. We observed that only minor differences in venom constitution are detected when comparing venom from newly arrived *Bothrops jararaca* and snakes born or kept for several years in captivity [36].

## Nursery Room

All newborn snakes, whether from females arriving pregnant from the wild or mating that occurred at LH, are kept until the 3^rd^ year of age in the nursery room. Young snakes up to one year old are fed every 15 days with pinkies, and from one year onwards are fed monthly with varying weights of rodents, depending on the size of the snake. At the age of three, young snakes are transferred to the maintenance rooms, where they are periodically milked.

## Clinical Analysis Laboratory

This room is equipped to perform hematological, biochemical and coproparasitological examinations to monitor the health status of the snakes maintained at the serpentarium, and to diagnose diseases and their prognosis. 

## Pathological Anatomy Laboratory

Snakes that die in the animal facility are sent to the Pathological Anatomy Laboratory, where necropsies are performed. Tissue fragments of all organs are collected for histopathological analysis. Necropsy examination is important to increase our knowledge about different species, determine macroscopic and microscopic alterations, as well as the etiologic agent and cause of death [23,37]. Our pathological anatomy laboratory is located outside the animal facility.

**Figure 7.**
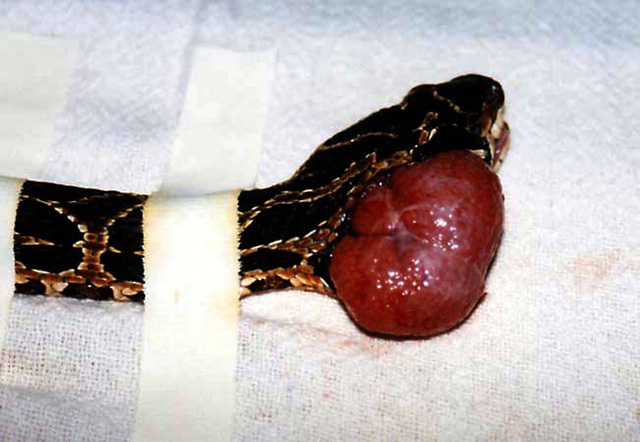
Venom gland tumor in an old *Bothrops alternatus*.

Necropsies are performed at LH since the 1990s, with 3050 necropsies performed until now. The major systems affected in most species are the digestive (gastritis and enteritis) and respiratory (caseous pneumonia) ones. Nowadays, with the increase in the longevity of our snakes, the presence of neoplasms has been increasingly common (Figure 7), as well as heart diseases.

## Traffic Flow Patterns

**Figure 8.**
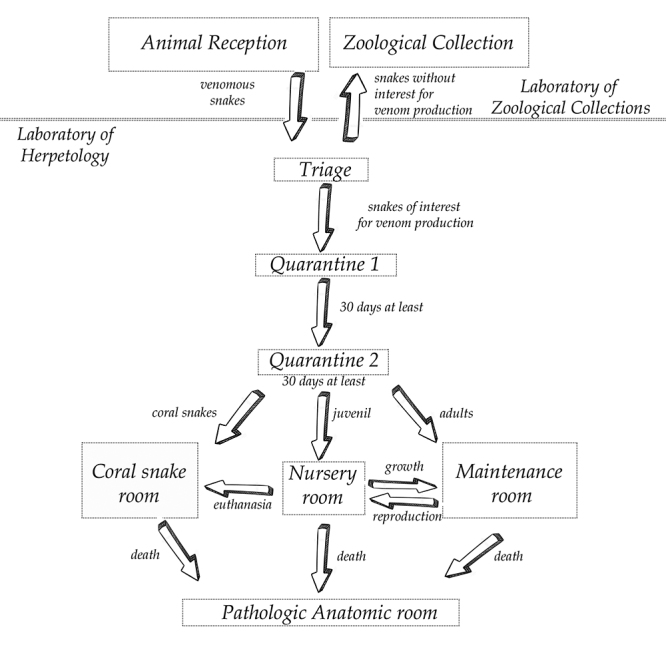
Traffic flow patterns at LH serpentarium.

One of the crucial hygienic-sanitary management in a serpentarium that must always be attended, is the traffic flow patterns, which must progress from the less contaminated areas (less potential of microbial contamination) to the highly contaminated areas (greater potential of microbial contamination), always in a one way direction (Figure 8). 

## Venom Production over the Years

The quality and quantity of venom produced are directly linked to the health status and welfare of the snakes. As maintenance conditions have been improving over the years, venom production (per snake) is also increasing, as can be seen in Table 1. The table shows that less venom was extracted in the period from 1908 to 1962. At this time, the snakes travelled by rail lines from different regions of Brazil in wooden boxes with water restriction, taking some days to arrive at the Institute. The snakes arrived stressed, dehydrated, and some venom was lost during physical restraint for milking procedure. Since the implantation of the indoors serpentarium in 1963, venom yielded/snake has been increasing due to the prophylactic and welfare improvements [8,9]. 

It is worth noting that the amount of venom extracted from *Crotalus durissus* and *Bothrops jararaca* newly arrived from nature (in the screening room) from 2005 to 2019 was 50% less than the volume extracted from snakes kept in captivity for at least 60 days (after quarantine period). In the genus *C. durissus*, the volume extracted in the screening room was very close to that reported for the outdoors serpentarium from 1908 to 1962. In the genus *B. jararaca*, the volume currently obtained from newly arrived individuals is twice that obtained in 1908-1962, but still 50% lower than the volume extracted from snakes kept in captivity for at least 60 days. 

## Conclusion

The maintenance of venomous snakes in captivity for venom production is essential. Annually, 20,000 snakebites occur in Brazil, and antivenom producers must meet the national demand for the supply of efficient antivenoms. This brief history showed that improvements made in the LH animal facility have increased the survival rate of the snakes over the years, as well as the amount and quality of venom produced. The most significant changes were the prophylactic measures including quarantine, unidirectional flow patterns, and the use of aprons and shoe covers when entering different areas of the serpentarium. The control of abiotic factors, such as temperature and humidity, according to the species kept in each room, increased the animals’ welfare. Furthermore, the LH has highly qualified personnel to handle, maintain and reproduce different species of snakes in captivity, besides performing clinical and anatomical pathology analysis, and venom research. Since 2000, the batches of venom produced at LH are submitted to biochemical analysis, such as protein concentration and dodecyl sulfate-polyacrylamide gel electrophoresis in reducing and non-reducing conditions; as well as toxicological analysis, such as LD_50_ (median lethal dose determination). These analyses are important to monitor the quality and effectiveness of the different venoms yielded.

A well-conducted serpentarium is a key element in the maintenance of venomous snakes for venom production. New techniques and efficient management should always be sought to improve snake welfare, the quality and amount of venom yielded, and the safety of those working directly with the venomous animals. All procedures carried out in a snake maintenance should be properly documented and scheduled.
